# miR-22 Controls *Irf8* mRNA Abundance and Murine Dendritic Cell Development

**DOI:** 10.1371/journal.pone.0052341

**Published:** 2012-12-14

**Authors:** Haiyan S. Li, Nathaniel Greeley, Naoshi Sugimoto, Yong-Jun Liu, Stephanie S. Watowich

**Affiliations:** 1 Department of Immunology, The University of Texas MD Anderson Cancer Center, Houston, Texas, United States of America; 2 Baylor Institute for Immunology Research, Baylor Research Institute, Dallas, Texas, United States of America; 3 The University of Texas Graduate School of Biomedical Sciences, Houston, Texas, United States of America; Oklahoma Medical Research Foundation, United States of America

## Abstract

MicroRNAs (miRNAs) have emerged as critical regulators of many cellular responses, through the action of miRNA-induced silencing complex (miRISC)- or miRNA ribonucleoprotein complex (miRNP)-mediated gene repression. Here we studied the role of miRNAs in the development of dendritic cells (DCs), an important immune cell type that is divided into conventional DC (cDC) and plasmacytoid DC (pDC) subsets. We found that miR-22 was highly expressed in mouse CD11c^+^ CD11b^+^ B220^−^ cDCs compared to pDCs, and was induced in DC progenitor cell cultures with GM-CSF, which stimulate CD11c^+^ CD11b^+^ B220^−^ cDC differentiation. Enforced overexpression of miR-22 during DC development enhanced CD11c^+^ CD11b^+^ B220^−^ cDC generation at the expense of pDCs, while miR-22 knockdown demonstrated opposite effects. Moreover, overexpression and knockdown of miR-22 showed significant effects on the mRNA abundance of *Irf8*, which encodes the transcription factor IRF8 that plays essential roles in DC development. Luciferase reporter assays confirmed that miR-22 binds directly to the 3′UTR of the mouse *Irf8* mRNA. Collectively, these results suggest that miR-22 targets *Irf8* mRNA for posttranscriptional repression and controls DC subset differentiation.

## Introduction

MicroRNAs (miRNAs) refer to a large family of short (usually 21–25 nucleotides in length), single-stranded non-coding RNA molecules [1]. They are found in various tissues across different species, and function as a posttranscriptional component of gene regulatory mechanisms in normal and malignant cell development. In vertebrates, most miRNAs are transcribed by RNA polymerase II from introns of protein-coding genes, then undergo sequential cleavages catalyzed by the nuclear RNase III enzymes Drosha and Dicer at primary and precursor miRNA levels [2]. The mature miRNA strand is incorporated into the so-called miRNA-induced silencing complex (miRISC) or miRNA ribonucleoprotein complex (miRNP), each containing an Argonaute protein, which will subsequently bind to the 3′UTR of target mRNAs based on seed nucleotide complementarity [1], [3], [4]. In mammalian cells, miRNAs repress gene expression mainly by inhibiting translation, or promote mRNA degradation via decapping or deadenylation. In addition, Argonaute 2 in miRISC or miRNP was reported to mediate mRNA silencing by its intrinsic RNA cleavage activity [5], [6].

miRNA expression is not only associated with distinct cell lineages, but also related to cellular differentiation/maturation stage. In the hematopoietic system, miRNAs regulate hematopoietic progenitors, T and B lymphocytes, as well as other immune cells [7]–[10]. For example, miR-155 is involved in homeostasis and function of the immune system, and disruption of miR-155 leads to defective dendritic cell (DC)-mediated antigen presentation, reduced numbers of germinal center B cells and Th2-biased T-cell responses with impaired Th1 and Th17 activation [2], [11]–[14]. By contrast, miR-223 is a negative regulator that is required to sustain granulocyte progenitors and granulopoiesis [15]–[17]. Experiments using conditional Dicer knockout mice suggest that Dicer-dependent miRNA expression is required for normal lymphocyte development [18]–[23].

In the current study, our goal was to identify miRNA regulators in DC lineage development. Two distinct DC populations, namely conventional DCs (cDC) and plasmacytoid DCs (pDC), have been classified in human and mouse. cDCs have been further subdivided on the basis of tissue localization, cell surface marker protein expression and function. Both cDC and pDC populations arise from common DC progenitors (CDPs, lin^−^ Flt3^+^ CD115^+^ CD117^−/lo^) in bone marrow, under control of key cytokines and lineage-restricted transcription factors (reviewed in [24] and [25]). Despite accumulating evidence indicating the important roles of miRNAs in hematopoiesis, little is known about their function in controlling DC development. By profiling miRNA expression in mouse pDCs and cDCs, we found that miR-22 is highly enriched in CD11c^+^ CD11b^+^ B220^−^ cDCs and suppressed in pDCs compared to its abundance in DC progenitors. We show that miR-22 binds to the 3′UTR of *Irf8* mRNA, which encodes IRF8, a transcription factor that is essential for pDC, CD8α^+^ cDC and CD103^+^ cDCs, but not for CD11c^+^ CD11b^+^ B220^−^ cDCs. miR-22 reduces *Irf8* mRNA amounts, suggesting posttranscriptional control of *Irf8* that may play a role in mediating DC lineage decisions.

## Materials and Methods

### Ethics Statement

C57BL/6NCr and congenic CD45.1^+^ mice were obtained from the National Cancer Institute or the Jackson Laboratory, and maintained in a specific pathogen free (SPF) facility at the University of Texas MD Anderson Cancer Center. All experimental procedures were approved and performed in accordance with Institutional Animal Care and Use Committee (IACUC) guidelines at MD Anderson Cancer Center (Protocol Number: 050205834).

### Isolation of DC progenitors, cDCs and pDCs

Murine bone marrow cells were obtained by flushing femurs and tibias with complete culture medium (RPMI supplemented with 10% FCS, 1% antibiotics and β-mercaptoethanol). Spleens were minced through nylon mesh and single cell suspensions were prepared. After lysis of RBCs, cells were stained with a rat anti-mouse lineage marker cocktail, including anti-CD3, CD19, Gr1, Ter-119 and F4/80, followed by staining with anti-rat IgG microbeads (Miltenyi). The lineage marker-positive cells were removed by passing cells through a magnetic column, according to the manufacturer's instructions (MACS). Negatively selected cell samples were further labeled with fluorescently-labeled antibodies against CD3, CD19, Gr1, Ter119, F4/80, Flt3, CD11c, CD11b and B220 (bone marrow) or CD11c, CD4, CD8α^−^ CD11b, and B220 (spleen) to sort DC progenitors (lineage marker (lin)^−^ Flt3^+^), bone marrow pDCs (CD11c^+^ CD11b^−^ B220^+^), splenic pDCs (CD11c^+^ CD11b^−^ B220^+^), splenic CD4^+^ cDCs (CD11c^+^ CD11b^+^ CD4^+^ CD8α^−^), splenic CD8α^+^ cDCs (CD11c^+^ CD11b^−^ CD4^−^ CD8α^+^) and splenic CD4^−^ CD8α^−^ cDCs (CD11c^+^ CD11b^+^ CD4^−^ CD8α^−^) by FACS, as described previously [26]. All antibodies were purchased from BD or eBioscience.

### Retro- and lentiviral constructs

To generate retroviral constructs that co-express miR-22 and GFP, sequences encoding the wild type (wt) or mutant (mut) miR-22 were released by XhoI and EcoRI digestion from pSuper-retroviral plasmids (kindly provided by Dr. Didier Picard, University of Geneva) [27] and inserted into a similarly digested pSuper-retroviral vector containing GFP. The GFP^+^ lentiviral vector-based anti-miR-22 or scrambled hairpin control, miRZip22 and MiRZip000, respectively, were kindly provided by Dr. Hidetoshi Tahara (Hiroshima University) [28]. Using these viruses, we routinely observed infection rates between 30–50% in Flt3L cultures and 70–90% in GM-CSF cultures, as judged by the frequency of GFP^+^ cells (data not shown).

### DC culture, viral transduction and flow cytometry

FACS-purified DC progenitors were cultured in the presence of complete RPMI medium containing Flt3L (100 ng/ml) or GM-CSF (50 ng/ml). In Flt3L cultures, 90% or greater of the CD11c^+^ CD11b^−^ B220^+^ pDCs express Siglec-H [26], while cDCs are a mixture of CD8α^+^-equivalent and CD8α^−^cDCs [29], [30]. By contrast, GM-CSF represses pDC production and mainly promotes the generation of CD11c^+^ CD11b^+^ B220^−^ (CD8α^−^) cDCs [31]. Two or three days later, cultured cells were collected for miRNA analysis by qPCR or subjected to viral transduction. To generate miR-22 overexpressing retroviruses, 293T cells were transfected with pSuper-GFP plasmids encoding wt or mut miR-22, in addition to the viral packaging plasmid pCL-Eco. To prepare anti-miR-22 lentiviruses, 293T cells were transfected with miRZip000 vector or miRZip22 plasmids, along with the viral packaging plasmids pPACKH1-GAG, pPACKH1-REV and pVSV-G. Virus-containing supernatants were collected at 48 and 72 h and used to infect cultured DC progenitor cells via a spin infection method (2300 rpm, 60 min). Two days following infection, cells were analyzed by flow cytometry using fluorescently-labeled antibodies against CD11c, CD11b, B220, MHC II, CD80 and CD86.

### Intravenous injection of DC progenitors

Lin^−^ Flt3^+^ DC progenitors were isolated from CD45.1^+^ congenic mice and cultured with Flt3L or GM-CSF as described (Methods, DC culture section). Two days later, cells were incubated with retroviruses expressing wt or mut miR-22, or with miRZip22 or miRZip000 lentiviruses for 8 hours. Progenitor cells were then washed with PBS, and injected i.v. into C57BL/6 mice (10^5^ cells/mouse/100 μl PBS). Recipient mice were sacrificed at d 7, and spleens were removed and analyzed for DC subset differentiation by flow cytometry.

### miRNA extraction, miRNA microarray profiling and qPCR analysis

Total RNA and miRNA were purified from freshly isolated DC progenitors, pDCs, cDCs or total bone marrow following culture in Flt3L or GM-CSF, as indicated in the figure legends, using the miRNeasy mini kit (Qiagen). The miRNA array profiling was performed at the Microarray Core Facility at MD Anderson Cancer Center, using Exiqon miRCURY LNA miRNA array 8.1. In addition, cDNAs were reverse transcribed by miScript Reverse Transcriptase and subjected to qPCR using QuantiTect SYBR Green PCR master mix (Qiagen). miRNA expression in qPCR was normalized to the internal control 5SrRNA [32] via the ΔC_T_ method [33]. The primer sequences for primary miR-22 are: sense 5′-CGAACAGCAGGGTGGATGAT-3′, antisense 5′-GGCAGAAAGCCTTGGGTTGT-3′.

### Dual luciferase reporter assays

The mouse *Irf8* 3′UTR (chromosome 8 genomic coordinates 120757176 to 120756694; encompassing 1521 bp downstream (3′) of the *Irf8* mRNA stop codon) was amplified by PCR with primers containing XbaI sites and cloned into the XbaI site of the PGL3-SV40 vector (Promega) downstream (3′) of the firefly luciferase region. Truncated *Irf8* 3′UTR sequences that lack of one or both predicted miR-22 seed regions were cloned via a similar approach, as indicated in the figure legends. D2SC/1 cells [34] were then transfected with the pGL3 constructs containing full length or truncated *Irf8* 3′UTRs, phRL-TK (encoding *Renilla* luciferase), and plasmids that overexpress or block miR-22, using Lipofectamine 2000 (Invitrogen). Firefly luciferase activity was determined 48 h after transfection, and normalized to the control *Renilla* level, using the Dual-Luciferase Reporter Assay System (Promega). Transcription of the firefly luciferase gene is constitutively controlled by the SV40 promoter, and is affected by the *Irf8* 3′UTR through the binding of miR-22.

### Statistical analysis

Data were compared by unpaired two-tailed Student's *t*-test using GraphPad Prism 5 (http://www.graphpad.com); *p* values <0.05 were considered to be statistically significant.

## Results

### miR-22 is differentially expressed in pDCs and cDCs

**Figure 1:**
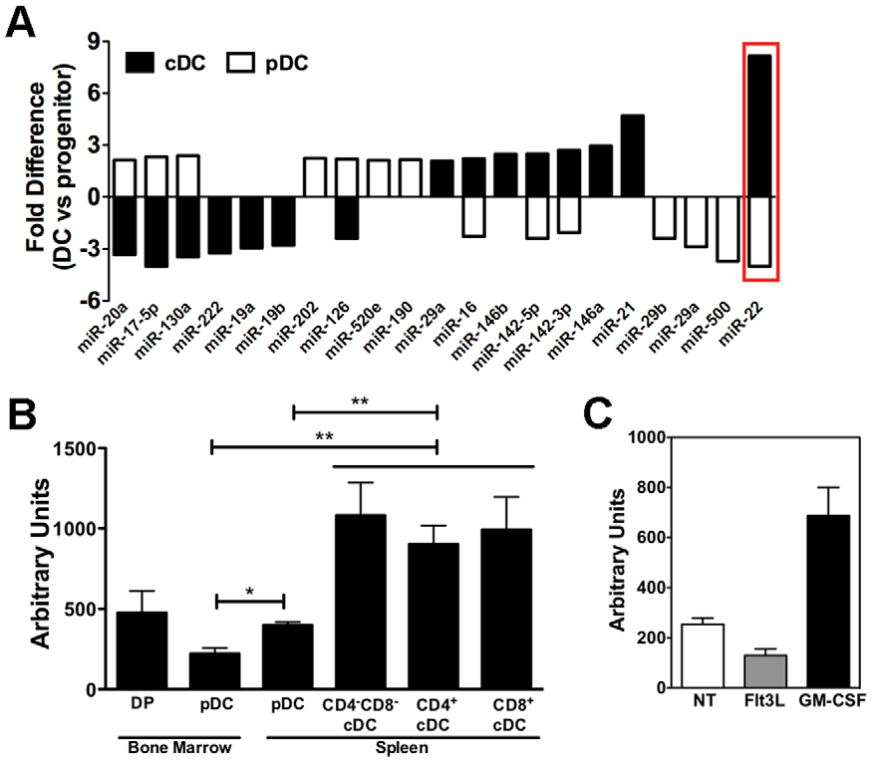
miRNA profiling in pDCs and cDCs. **A.** Total RNA, including miRNA, was isolated from FACS-purified bone marrow lin^−^ Flt3^+^ progenitors, bone marrow pDCs and splenic CD11c^+^ CD11b^+^ B220^−^ cDCs and analyzed by miRNA array. miRNA expression in pDCs and cDCs was normalized to expression in bone marrow lin^−^ Flt3^+^ progenitors, and presented as mean ± SD of 2 independent experiments. **B.** miR-22 expression in FACS-purified lin^−^ Flt3^+^ DC progenitors (DP), pDCs or individual cDC subsets, isolated from bone marrow or spleen as indicated, was analyzed by qPCR. **C.** lin^−^ Flt3^+^ DC progenitors were cultured in the presence of Flt3L or GM-CSF for 72 h; cells were analyzed for miR-22 expression by qPCR. **B–C.** Data are normalized to 5SrRNA, and shown as mean ± SD of 3 independent experiments.

To characterize miRNA expression in DCs, we isolated total RNA including miRNA from bone marrow pDCs and splenic CD11c^+^ CD11b^+^ B220^−^ cDCs and hybridized labeled RNAs to Exiqon miRCURY LNA miRNA array 8.1. We also analyzed RNA from lineage-negative, fms-like tyrosine kinase receptor-3 (Flt3)-positive (lin^−^ Flt3^+^) DC progenitors in bone marrow to provide a comparison for potential developmental changes in miRNA expression. We profiled miRNA samples in 2 independent experiments and found highly consistent results showing 21 miRNAs that were differentially expressed in pDCs versus CD11c^+^ CD11b^+^ B220^−^ cDCs (Fig. 1A). For example, miR-22, miR-142-3p and miR-142-5p were upregulated in CD11c^+^ CD11b^+^ B220^−^ cDCs and downregulated in pDCs relative to progenitor expression levels, while miR-20a, miR-17-5p and miR-130a showed the reverse pattern. miR-22 showed the most remarkable difference between the two DC subsets (Fig. 1A), and thus we selected it for further analysis. To confirm differential miR-22 expression in DCs and their progenitors, we performed qPCR analysis on RNA samples from cell populations isolated from bone marrow or spleen. We found that miR-22 is highly enriched in all splenic cDC subsets, including CD4^+^, CD8α^+^ and CD4^−^ CD8α^−^ cDCs, while being expressed at relatively lower amounts in pDCs from bone marrow or spleen (Fig. 1B). To examine whether miR-22 is regulated during DC development, we cultured lin^−^ Flt3^+^ DC progenitors ex vivo with Flt3L or GM-CSF, conditions that promote, respectively, the differentiation of pDCs and cDCs in an approximate 1:1 ratio, or selective cDC differentiation including CD11c^+^ CD11b^+^ B220^−^ cDCs (reviewed in [25]). We found that miR-22 was highly induced in GM-CSF cultures, while slightly repressed in Flt3L cultures (Fig. 1C). These results collectively suggest that miR-22 expression is regulated during DC development, with upregulation in CD11c^+^ CD11b^+^ B220^−^ cDCs and suppression in pDCs compared to DC progenitors.

### miR-22 affects DC subset differentiation and maturation

**Figure 2:**
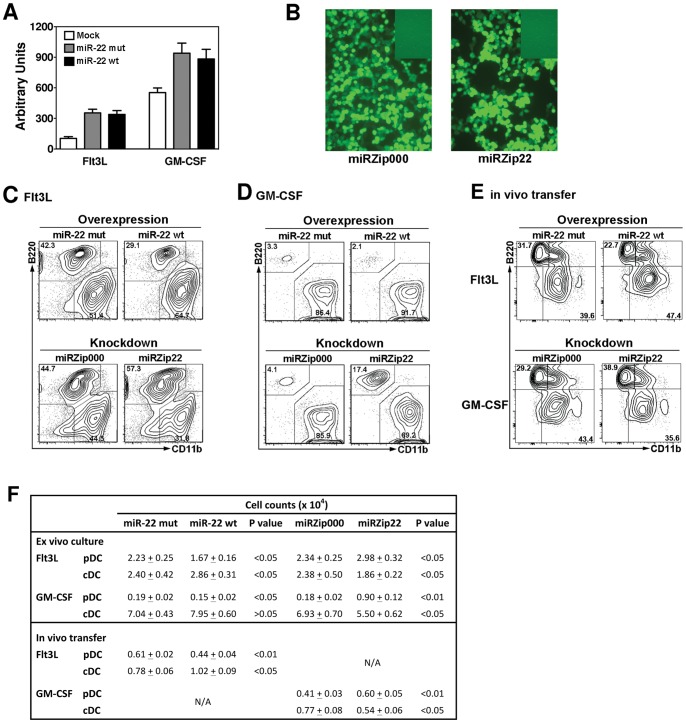
miR-22 facilitates cDC generation while suppressing pDC development. **A.** lin^−^ Flt3^+^ DC progenitors were cultured with Flt3L or GM-CSF for 2 days, and infected with retroviruses expressing wt or mut miR-22 or with empty vector (mock), as indicated. Two days after infection, cells were collected and analyzed for miR-22 expression by qPCR. Data were normalized to 5SrRNA, and shown as mean ± SD of 3 independent experiments. **B.** 293T cells were transfected with miR-22-knockdown plasmid miRZip22 or miRZip000 control vector, together with pPACK packaging plasmids. GFP expression was analyzed by fluorescence microscopy 2 days following transfection. The small rectangle on the right upper corner shows the uninfected control. **C–D.** 10^5^ lin^−^ Flt3^+^ DC progenitors were cultured with Flt3L (**C**) or GM-CSF (**D**). Two days later, cells were infected with retroviruses expressing wt or mut miR-22 (overexpression), or with miRZip22 or miRZip000 lentiviruses (knockdown). Two days after infection, cells were collected and analyzed for DC generation by FACS. Representative flow data within the gated GFP^+^ CD11c^+^ population from 3 independent experiments are shown in **C–D**. **E.** 10^5^ lin^−^ Flt3^+^ DC progenitors from CD45.1^+^ congenic mice were cultured with Flt3L (upper panel) or GM-CSF (lower panel) for 2 d, followed by viral infection to overexpress or knockdown miR-22 as described in **C–D**. Eight hours after infection, cells were injected i.v. into C57BL/6 mice (CD45.2^+^). Six days later, spleens were collected and analyzed for DC populations by FACS. Representative flow data are shown within the gated CD45.1^+^ CD11c^+/lo^ cells. **F.** The absolute number of pDCs and cDCs from the in vitro (**C, D**) or in vivo (**E**) assays were determined by enumeration. The mean ± SD of absolute DC numbers (3–5 experiments/group) are shown.

To examine the role of miR-22 in DC development and function, we employed GFP-encoding retro- and lentiviral constructs to enforce its overexpression or knockdown. We confirmed that wt or seed nucleotide mut miR-22 was overexpressed by pSUPER retroviral vectors in Flt3L- or GM-CSF-cultured lin^−^ Flt3^+^ DC progenitors, relative to mock-infected controls, using qPCR analysis (Fig. 2A). The enhanced expression of wt and mut miR-22 observed in DC progenitors cultured with GM-CSF versus Flt3L is likely due to higher endogenous miR22 levels in GM-CSF conditions or suppressive effects of Flt3L (Fig. 1C), as indicated by the mock-infected controls. In addition, we found that the miRZip lentiviral vector-based anti-miR-22, which antagonizes miR-22 function by blocking binding to target mRNAs, was expressed in >90% transfected 293 T cells (Fig. 2B), indicating efficient generation of anti-miR-22 lentiviruses.

To investigate whether miR-22 controls pDC and/or cDC differentiation, we cultured lin^−^ Flt3^+^ DC progenitors in Flt3L or GM-CSF for 2 days, infected cells with miR-22 overexpression or knockdown viruses, and analyzed the development of CD11c^+^ CD11b^−^B220^+^ pDCs and CD11c^+^ CD11b^+^ B220^−^ cDCs within the GFP^+^ population. We found that overexpression of wt miR-22 enhanced the production of CD11c^+^ CD11b^+^ B220^−^ cDCs and inhibited generation of pDCs in response to Flt3L, as evidenced by corresponding changes in the frequency (Fig. 2C upper panels) and absolute number (Fig. 2F) of each DC subset. A similar trend was observed in GM-CSF cultures (Fig. 2D upper panels; 2F); however, the changes in cDC numbers did not reach statistical significance, which is most likely due to the predominant role of GM-CSF in driving cDC production and repressing pDCs [31]. By contrast, when miR-22 was blocked by miRZip22, pDC development was enhanced and CD11c^+^ CD11b^+^ B220^−^ cDC generation was suppressed in both Flt3L and GM-CSF cultures (Fig 2C–D lower panels; 2F). To determine whether miR-22 regulates DC development in vivo, Flt3L- or GM-CSF-cultured CD45.1^+^ DC progenitors were infected with miR-22-overexpression or knockdown viruses, or the appropriate controls, then transferred into CD45.2^+^ recipient mice. For these assays, we chose the cytokine conditions that displayed the optimal differences in DC subset development ex vivo (Fig. 2C and D). Six days after adoptive transfer, spleens were collected and analyzed for CD45.1^+^ CD11c^+^ DC development. Consistent with the ex vivo results, overexpression of miR-22 in Flt3L-stimulated DC progenitors significantly enhanced CD11c^+^ CD11b^+^ B220^−^ cDC production while repressing pDCs (Fig. 2E upper panel). Moreover, miR-22 knockdown in progenitors exposed to GM-CSF had the opposite effects (Fig. 2E lower panel). Collectively, these data suggest that miR-22 influences DC subset development.

**Figure 3:**
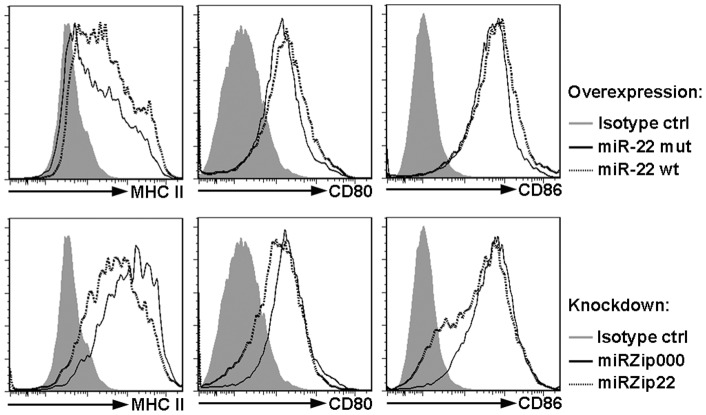
miR-22 promotes MHC and costimulatory molecule expression on cDCs. Total bone marrow cells were cultured with Flt3L or GM-CSF for 3 days, followed by infection with retroviruses expressing wild type (wt) or mutant (mut) miR-22. Two days after infection, GFP^+^ cells were gated and analyzed for DC markers by flow cytometry. MHC II, CD80 and CD86 expression on GFP^+^ CD11c^+^ CD11b^+^ B220^−^ cDCs is shown. Results represent 3 independent experiments.

Since miR-22 was predominantly expressed in CD11c^+^ CD11b^+^ B220^−^ cDCs compared to pDCs (Fig. 1), we examined its function in cDCs derived from GM-CSF cultures. We used GFP-encoding miR-22 overexpression or knockdown vectors to manipulate miR-22 expression, and analyzed MHC class II (MHC II), CD80 and CD86 expression on GFP^+^ CD11c^+^ CD11b^+^ B220^−^ cDCs. We found that miR-22 overexpression in cDCs upregulated MHC II expression, relative to MHC II amounts observed with mut miR-22 overexpression (Fig. 3, upper panels). We also observed a modest increase in CD80 and CD86 expression upon miR-22 overexpression (Fig. 3, upper panels). By contrast, we found that miR-22 knockdown suppressed surface presentation of MHC II, CD80 and CD86 compared to cells infected with the miRZip000 control vector (Fig. 3, lower panels). Importantly, since we used comparisons with cells infected with mut or empty vector in these assays, our approach ruled out the possibility that changes in MHC II, CD80 and CD86 were related to viral infection. Thus, our results suggest that miR-22 directly or indirectly enhances cell surface expression of MHC II and costimulatory molecules in CD11c^+^ CD11b^+^ B220^−^ cDCs.

### miR-22 affects Irf8 mRNA through direct binding to its 3′UTR

**Figure 4:**
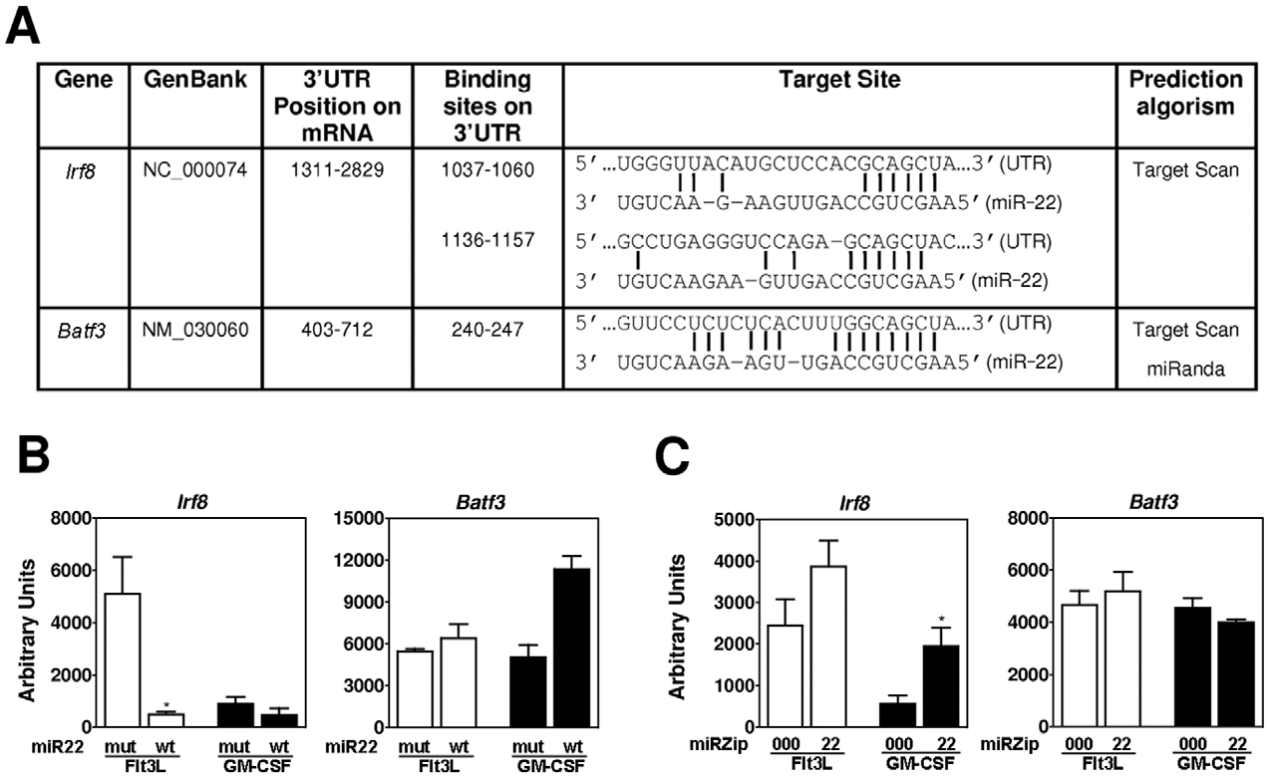
miR-22 regulates *Irf8* mRNA abundance. **A**. Summary of potential miR-22 target sites in the 3′-UTR of *Irf8* and *Batf3* mRNAs. **B–C**. lin^−^ Flt3^+^ progenitors were cultured for 3 days with Flt3L or GM-CSF, as indicated. Cells were infected with retroviral vectors expressing wt or mut miR-22 (**B**) or lentiviral vectors expressing control (miRZip000) or anti-miR-22 (miRZip22) as indicated (**C**). Two days after infection, the relative expression of *Irf8* and *Batf3* mRNA was determined by qPCR and normalized to 5S rRNA amounts. Data are shown as mean ± SD of 3 independent experiments.

Individual miRNAs can target hundreds of mRNAs through complementarity of the seed region nucleotides [1], [3], [4]. To identify potential targets of miR-22, we performed an algorithm-based prediction using two widely utilized software programs (Target Scan and MiRanda). Among more than 2000 potential target genes (not shown), we selected *Irf8* and *Batf3* for further study, as these two genes encode transcription factors critical for DC development and function [35]–[43]. We found that the 3′ UTR of *Irf8* mRNA (∼1500 bp) contains 2 putative miR-22 binding sites, while the 3′UTR of *Batf3* (∼400 bp) contains 1 potential binding site (Fig. 4A). To assess whether *Irf8* and *Batf3* are targets of miR-22, we quantified their mRNA amounts in lin^−^ Flt3^+^ DC progenitors following enforced overexpression of wt or mut miR-22, or in response to miRZip22-mediated miR-22 knockdown. We observed a dramatic (∼10-fold) reduction of *Irf8* mRNA amounts upon miR-22 overexpression in lin^−^ Flt3^+^ DC progenitors cultured with Flt3L (Fig. 4B). By contrast, we found that *Irf8* was expressed at lower amounts in lin^−^ Flt3^+^ DC progenitors cultured with GM-CSF compared to Flt3L, and miR-22 overexpression did not appear to affect *Irf8* expression in GM-CSF conditions (Fig. 4B). Consistent with this, we observed that miRZip22-mediated miR-22 knockdown enhanced *Irf8* mRNA levels in GM-CSF-cultured progenitors in comparison to miRZip000 control, while miR-22 knockdown had only modest effects upon the already high amounts of *Irf8* mRNA in Flt3L cultures (Fig. 4C). miR-22 overexpression or knockdown did not appear to affect *Batf3* mRNA, with the exception of increased *Batf3* mRNA amounts in GM-CSF cultures with miR-22-overexpression (Fig. 4C). These data indicate that miR-22 specifically reduces *Irf8* mRNA abundance, potentially via enhanced mRNA degradation.

**Figure 5:**
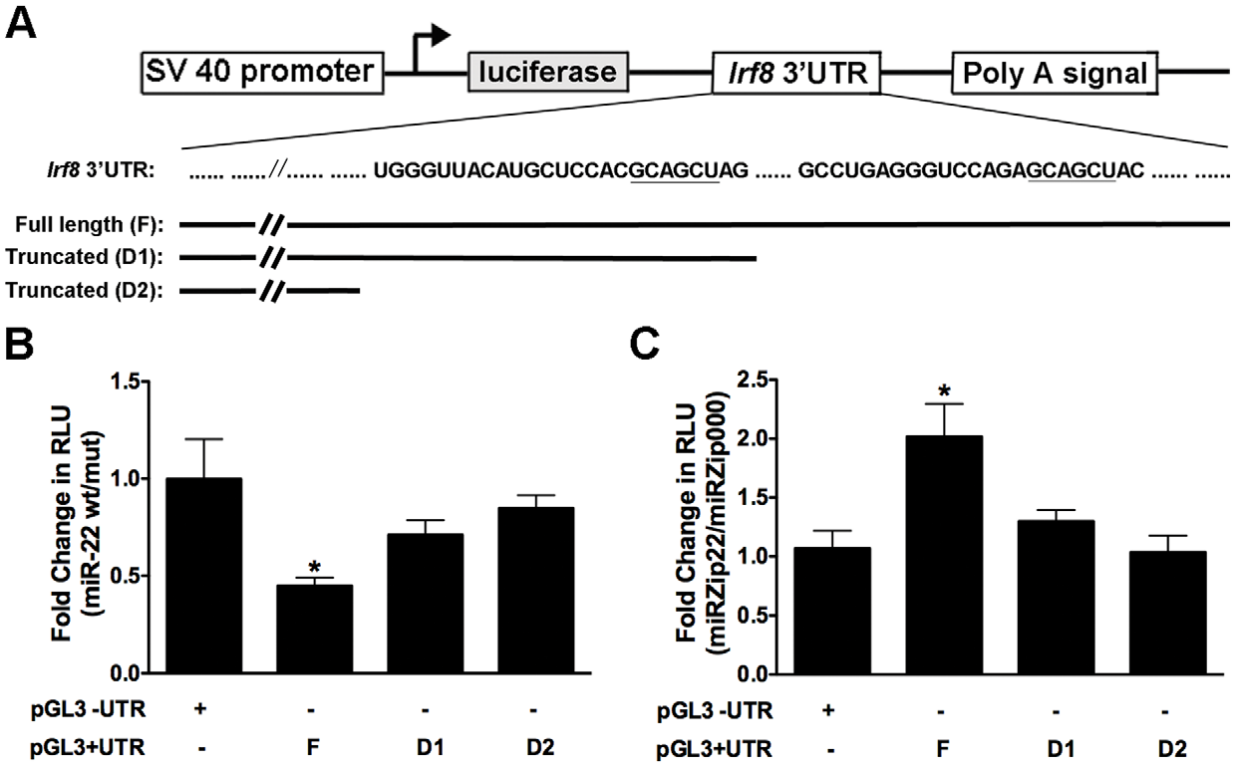
miR-22 targets the 3′UTR of *Irf8*. **A**. Schematic diagram of the pGL3 luciferase reporter constructs containing full length or truncated versions of the 3′-UTR of *Irf8* mRNA. **B–C**. D2SC/1 cells were transfected using Lipofectamine 2000 with pGL3 constructs described in **A**, phRL-TK, and miR-22 overexpression (**B**) or knockdown (**C**) plasmids. The ratio of *firefly: Renilla* luciferase light units (RLU) was determined 48 h after transfection using the Dual-Luciferase Reporter Assay System, and the fold change in RLU was calculated as indicated on y-axis. Results are presented as mean ± SD of 3 independent experiments.

To address whether *Irf8* mRNA is a direct target of miR-22, we performed luciferase reporter assays in a cDC cell line, D2SC/1 [34]. For these experiments we generated a pGL3 reporter construct that contains the full length *Irf8* 3′ UTR downstream of the open reading frame of firefly luciferase (Fig. 5A and data not shown). We also generated 2 truncated *Irf8* 3′ UTR constructs, which lack one or both of the putative miR-22-binding sites, to better evaluate the function of these regions (Fig. 5A). The firefly luciferase activity of all three constructs is controlled by the SV40 promoter, and the addition of *Irf8* 3′UTR with or without the miR-22 seed region(s) allows us to monitor the changes in reporter activity fine-tuned by miR-22 binding. We found that wt miR-22 inhibited reporter activity of the construct containing the full length *Irf8* 3′UTR, relative to effects of the mut miR-22 on reporter activity (Fig. 5B). Consistently, knockdown of miR-22 function by miRZip22 induced an approximate 2-fold increase in the activity of the full length *Irf8* 3′UTR reporter compared to effects of the miRZip000 control on reporter function (Fig. 5C). These results suggest that miR-22 negatively regulates transcription of the firefly luciferase reporter gene in the presence of the *Irf8* 3′UTR. Moreover, we demonstrated that miR-22-binding sites in the *Irf8* 3′UTR are required for miR-22-mediated repression of reporter activity, as the truncated *Irf8* 3′UTR reporter constructs that lack one or both miR-22-binding sites were either partially or completely refractory to miR-22-mediated regulation in both overexpression and knockdown settings (Fig. 5B, 5C). These results collectively indicate that miR-22 interacts with miR-22 seed regions in the *Irf8* 3′UTR and mediates *Irf8* mRNA repression.

## Discussion

Despite the fact that murine pDCs and cDCs originate from a common DC progenitor, they require distinct growth factors and molecular cues for their lineage specification and development, which results in the acquisition of their divergent morphology and function. We report here that miR-22 enhances the generation of CD11c^+^ CD11b^+^ B220^−^ cDCs from DC progenitors in vivo and in vitro and stimulates the mature phenotype of this subset, consistent with abundant miR-22 amounts observed in CD11c^+^ CD11b^+^ B220^−^ cDCs relative to pDCs or DC progenitors. Furthermore, we found that miR-22 directly regulates *Irf8* mRNA amounts in DCs, potentially via targeting *Irf8* mRNA for destruction. To our knowledge, this is the first study demonstrating the role of miR-22 in DC development, suggesting miR-22 involvement in normal hematopoiesis and cell differentiation.

Numerous miRNAs have been shown to regulate immune cell development and function, such as miR155, miR-223 and miR-181 (reviewed in [7], [44], [45]). These miRNAs exert their roles by fine-tuning the expression levels of critical transcription factors and genes involved in mature cell activities. While *Batf3* and *Stat5* were identified in our software analyses as putative miR-22 targets, we were unable to obtain evidence for their direct regulation by miR-22. By contrast, here we show that miR-22 targets murine *Irf8* mRNA directly through a complementary seed region in the *Irf8* 3′UTR. We have previously demonstrated that *Irf8* is inducible by the DC growth factor Flt3L, whereas the cytokines GM-CSF and IFN-α directly repress or stimulate *Irf8* transcription via the transcriptional regulators STAT5 or STAT1, respectively [31], [33]. The GM-CSF-STAT5- and miR-22-mediated regulatory pathways may work cooperatively at transcriptional and post-transcriptional levels, respectively, to precisely control the amount of *Irf8* mRNA expression during DC subset differentiation. By contrast, human *IRF8* 3′UTR does not appear to contain a miR-22 seed region and miR-22 is not differentially expressed in human pDCs and cDCs (data not shown). However, inspection of the human *IRF8* 3′UTR revealed potential target sites for multiple miRNAs, including miR-130a, miR-19a and miR-19b, which are differentially regulated in mouse pDCs and cDCs (results herein). These, and other miRNAs that are specifically regulated in humans, may be involved in controlling human *IRF8* expression in analogous but independent regulatory pathways from the miR-22-mediated mechanism described herein.

IRF8 is a transcription factor that plays an essential role in controlling the development and cytokine secretion of several murine DC subsets, including pDCs, lymphoid CD8α^+^ cDCs and non-lymphoid CD103^+^ DCs [37], [38], [40], [42], [46], [47]. The role of IRF8 in DC development is conserved in humans as point mutations (T80A, K108E) in the IRF8 DNA-binding domain are found in individuals with immunodeficiency accompanied by impaired DC subset production [36]. By contrast, IRF8 is dispensable for homeostatic production of murine CD11c^+^ CD11b^+^ B220^−^ cDCs, a population that mainly contains CD8α^−^ cDCs [31], [38]. Interestingly, we did not observe a significant difference in miR-22 amounts in splenic CD4^+^, CD8α^+^ or CD4^−^ CD8α^−^ cDC subsets, although these populations have differential requirements for IRF8 [35], [38]. These results suggest that the developmental effects of miR-22, potentially via *Irf8* regulation, may occur at pre-cDC population prior to cDC subset diversification.

miR-22 was thought to be a ubiquitously expressed miRNA [48], yet we show here that miR-22 is highly enriched in murine CD11c^+^ CD11b^+^ B220^−^ cDCs in comparison to pDCs, suggesting important regulation of miR-22 expression occurs in the hematopoietic system. This distinct expression pattern is consistent with miR-22 function in DC lineage differentiation, as our overexpression and knockdown of miR-22 supports the idea that miR22 promotes CD11c^+^ CD11b^+^ B220^−^ cDC production and inhibits pDC development. In addition, we found that miR-22 modestly enhances MHC class II and costimulatory molecule expression in CD11c^+^ CD11b^+^ B220^−^ cDCs, suggesting it also affects genes that are involved cDC antigen presentation, a major function for the cDC subsets. miR-22 has been previously implicated in regulating histone modifications [49], [50], as well as tumorigenesis by controlling tumor cell proliferation, migration/invasion and apoptosis [51]–[54]. More importantly, miR-22 has been characterized in multiple signaling pathways that play critical roles in hematopoiesis and cellular function, including NFκB [55], PTEN/AKT [56], [57] and estrogen receptor [58] responses. These pathways are also involved in DC development and function. For examples, PTEN was identified as a negative regulator for CD8α^+^ and CD103^+^ DCs downstream of Flt3 signaling [59], while NFκB and estrogen receptor are critical for signals elicited by pattern recognition receptors expressed on DCs [60], [61]. As one miRNA may have hundreds of target genes, the regulatory function of miR-22 in these signaling cascades may contribute to its effects on DC development and function.

In addition to our results that identify miR-22 as a negative regulator of the DC transcription factor IRF8 by controlling *Irf8* mRNA abundance, recent studies by others have shown that miR21, miR34a, miR-221 and miR-222 are differentially expressed in pDCs and cDCs, and play a role in DC differentiation via inhibitory functions on Jag1, *Wnt1*, and possibly the pDC master regulator *Tcf4* (E2–2), respectively [62]–[66]. Among these, Kuipers *et* *al*. reported that miR-222 is expressed at 2.5-fold higher levels in cDCs versus pDCs purified from bone marrow cell cultures supplemented with Flt3L and SCF [62]. By contrast, our miRNA array profiling data revealed that miR-222 is expressed at lower levels in freshly isolated splenic cDCs in comparison to bone marrow DC progenitors or pDCs (Fig. 1A). This discrepancy may reflect the regulation of miRNA expression by cytokines, the different cell purification strategies used, or possible differences between microRNA array and miRNA measurements. Nonetheless, the available evidence suggests miRNAs serve as another layer of gene regulation that exerts profound influence on DC subset specification. In future work, it will be important to understand how miRNAs are regulated within DCs as well as the effects they exert on DC development and function. This information may prove valuable in redirecting DCs for use in clinical applications.
